# Supplementary material for: Spatial control of mitochondrial retrograde signaling by nuclear pore-associated contact sites
Source: bioRxiv. 2026 Jul 24:2026.07.23.740218. Preprint. [Version 1] doi: 10.64898/2026.07.23.740218 (PMC13419819; doi:10.64898/2026.07.23.740218)
Supplement: 1 [file NIHPP2026.07.23.740218v1-supplement-1.pdf]

bioRxiv preprint doi: https://doi.org/10.64898/2026.07.23.740218; this version posted July 24, 2026. The copyright holder for this preprint (which was not certified by peer review) is the author/funder, who has granted bioRxiv a license to display the preprint in perpetuity. It is made available under a CC-BY 4.0 International license.

Supplemental Figure S1. Kinetics of GPS2 nuclear accumulation in response to Complex I inhibition by Rotenone treatment, nuclear envelope integrity, and mitochondria–ER contact sites. Related to Figure 1.

bioRxiv preprint doi: https://doi.org/10.64898/2026.07.23.740218; this version posted July 24, 2026. The copyright holder for this preprint (which was not certified by peer review) is the author/funder, who has granted bioRxiv a license to display the preprint in perpetuity. It is made available under a CC-BY 4.0 International license.

(A) Representative immunofluorescence images of 3T3-L1 cells treated for 3 hours with Rotenone 1µM or DMSO control, stained for DAPI (blue), GPS2 (red), and TOMM70 (green). Right, quantification of nuclear GPS2 intensity (MGV per nucleus), DMSO versus ROT (3 h). Statistical analysis of three independent experiments was performed using an unpaired t-test. Statistical significance was defined as follows: p-value 0.1234 (ns), 0.0332 (*), 0.0021 (**), 0.0002 (***), and <0.0001 (****).

(B) Representative immunofluorescence images of U2OS cells treated with DMSO, 1 hour Rotenone, or 2 hours Rotenone, stained for DAPI (blue), GPS2 (magenta), and ATP5A (green); single-channel GPS2 (gray scale) shown. The scale bar represents 20 µm. Data points in the adjacent graph represent individual nuclei for the evaluation of mean nuclear GPS2 intensity. For DMSO, *n*= 25; 1 hour Rotenone, *n*=21; 2 hours Rotenone, *n*=22. P values were calculated using one-way ANOVA test (* represents p< 0.05 and ns stands for non-significant). Data are presented as mean ± SEM from three independent experiments.

(C) Lamin immunofluorescence demonstrating that nuclear envelope structure is not grossly altered by mitochondrial stress. Top, merged lamin A/C and DAPI images. Bottom, identically processed lamin A/C images displayed to improve visualization; the same convolution and Gaussian-blur parameters were applied to all conditions. The scale bar represents 20 µm.

(D) Short-range Mitochondria–ER contact sites were visualized by the SPLICS-P2A$^{ER\text{-}MT}$ reporter following FCCP treatment, showing no stress-induced increase in perinuclear mito-ER contacts. Representative channels show SPLICS-GFP (green), DAPI (blue), and ATP5A (red). Scale bar: 20 µm.

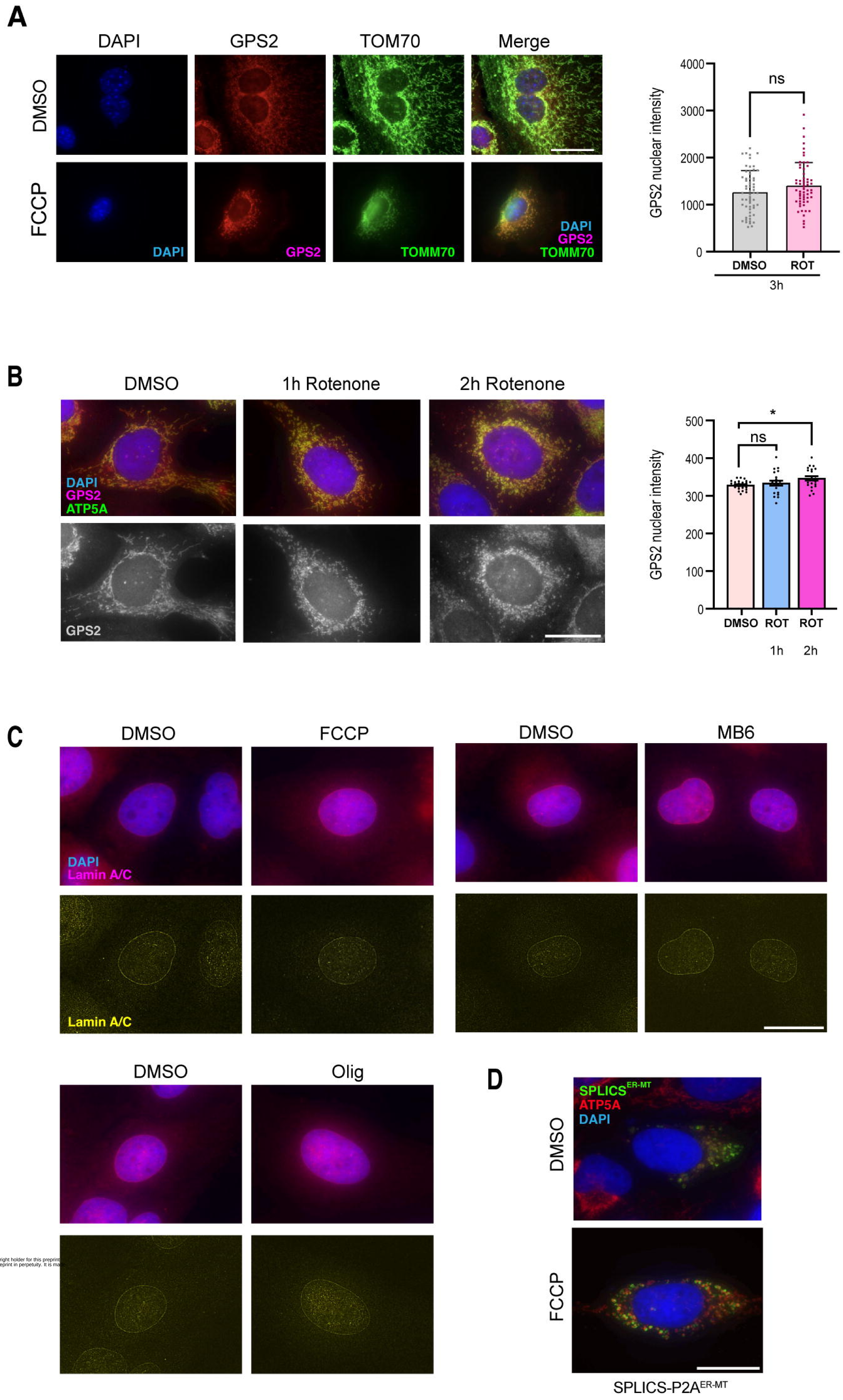

bioRxiv preprint doi: https://doi.org/10.64898/2026.07.23.740218; this version posted July 24, 2026. The copyright holder for this preprint (which was not certified by peer review) is the author/funder, who has granted bioRxiv a license to display the preprint in perpetuity. It is made available under a CC-BY 4.0 International license.

Mitra et al, 2026 Supp Figure 1
